# Autoimmune Thyroid Diseases in Patients Treated with Alemtuzumab for Multiple Sclerosis: An Example of Selective Anti-TSH-Receptor Immune Response

**DOI:** 10.3389/fendo.2017.00254

**Published:** 2017-09-28

**Authors:** Mario Rotondi, Martina Molteni, Paola Leporati, Valentina Capelli, Michele Marinò, Luca Chiovato

**Affiliations:** ^1^Unit of Internal Medicine and Endocrinology, Laboratory for Endocrine Disruptors, ICS-Maugeri IRCCS, University of Pavia, Pavia, Italy; ^2^Department of Internal Medicine and Therapeutics, and Department of Medical and Surgical Sciences, University of Pavia, Pavia, Italy; ^3^Endocrinology Unit I, Department of Clinical and Experimental Medicine, University of Pisa, University Hospital of Pisa, Pisa, Italy

**Keywords:** Graves’ disease, Alemtuzumab, multiple sclerosis, autoimmune thyroid disease, reconstitution syndrome

## Abstract

Alemtuzumab, a humanized anti-CD52 monoclonal antibody, is approved for the treatment of active relapsing-remitting multiple sclerosis (MS). Alemtuzumab induces a rapid and prolonged depletion of lymphocytes from the circulation, which results in a profound immuno-suppression status followed by an immune reconstitution phase. Secondary to reconstitution autoimmune diseases represent the most common side effect of Alemtuzumab treatment. Among them, Graves’ disease (GD) is the most frequent one with an estimated prevalence ranging from 16.7 to 41.0% of MS patients receiving Alemtuzumab. Thyrotropin (TSH) receptor (R)-reactive B cells are typically observed in GD and eventually present this autoantigen to T-cells, which, in turn, secrete several pro-inflammatory cytokines and chemokines. Given that reconstitution autoimmunity is more frequently characterized by autoantibody-mediated diseases rather than by destructive Th1-mediated disorders, it is not surprising that GD is the most commonly reported side effect of Alemtuzumab treatment in patients with MS. On the other hand, immune reconstitution GD was not observed in a large series of patients with rheumatoid arthritis treated with Alemtuzumab. This negative finding supports the view that patients with MS are intrinsically more at risk for developing Alemtuzumab-related thyroid dysfunctions and in particular of GD. From a clinical point of view, Alemtuzumab-induced GD is characterized by a surprisingly high rate of remission, both spontaneous and after antithyroid drugs, as well as by a spontaneous shift to hypothyroidism, which is supposed to result from a change from stimulating to blocking TSH-receptor antibodies. These immune and clinical peculiarities support the concept that antithyroid drugs should be the first-line treatment in Alemtuzumab-induced Graves’ hyperthyroidism.

## Alemtuzumab as an Immunomodulating Drug

Alemtuzumab is a humanized monoclonal antibody that has been approved for the treatment of active relapsing-remitting (RR) multiple sclerosis (MS) (1, 2). As a main pharmacologic action, Alemtuzumab targets the cell-surface antigen CD52. CD52 is a cell-surface glycoprotein with a still poorly understood function. CD52 is expressed on the surface of more than 95% T and B cells, of monocytes and of some dendritic cells, and, although to a lesser extent, even on natural killer cells and other leukocytes (3). The binding of Alemtuzumab to lymphocytes induces cellular lysis leading to their rapid and prolonged depletion from the circulation (4).

The acute immuno-suppressive effect of Alemtuzumab is followed by the homeostatic reconstitution of immune cells. Typically, monocytes and B cells recover first, followed by CD4+ T cells. Changes in lymphocyte subsets result in an increased number of T regulatory (Treg) cells and of memory T and B lymphocytes; an increased production of anti-inflammatory cytokines also occurs (5). These events produce a profound rebalance of the immune system (6, 7).

Circulating lymphocytes disappear within a few minutes after the administration of Alentuzumab. B cells recover within 3 months and a dominance of mature naïve cells (CD19+ CD23+ CD27−) over the memory B cells occurs. CD4+ T cell counts are restored after 35 months, while CD8+ T cell counts are restored after 20 months. The faster recovery of the latter subset of T cells might be related to the development of autoimmune diseases (8). For at least 9 months after the administration of Alemtuzumab, most circulating T cells are represented by effector memory CD4+ and CD8+ cells. Baker et al. recently described the kinetics of lymphocyte subset reconstitution after Alemtuzumab (9). After depletion, B cells repopulated much more rapidly than T cells in general and Treg in particular (9). In this scenario, the reconstitution of B cells without adequate regulatory control by T cells may explain the high prevalence of post-Alentuzumab autoimmunity (9, 10).

Alentuzumab-induced lymphocytopenia is followed by the homeostatic growth of T cells, which is stimulated by the T cell receptor–self peptide complex. The process results in the appearance of an oligoclonal cell population, which tends to autoreactivity. New T cell populations have typical aspects of memory T cells, such as lower dependency to co-stimulation, need for lower antigen doses than naïve cells, and faster secrection of inflammatory cytokines when re-stimulated (6–8). The above described immune derangements lead to a reduced self-tolerance. In most patients, the proliferation of regulatory lymphocytes is unable to prevent autoimmune deseases, possibly because T cells undergo a faster homeostatic growth, which increases their resistance to regulation (8). Patients who developed autoimmunity after Alentuzumab treatment also show high basal levels of IL-21, a cytokine which increases the growth of auto-reactive T cells. In general, the cytokine expression is skewed to the Th2 profile, in agreement with the high B cell counts (11, 12).

Innate immunity is not affected, and no clinically relevant infection appears after Alentuzumab treatment. This can be due to the maintenance/growth of memory T cells.

The above described immunomodulating actions of Alemtuzumab are responsible for its favorable effects in patients with RRMS (13), but also explain the high prevalence of Alemtuzumab-induced autoimmunity. The latter event received great concern and clinical trials aimed at evaluating potential preventive measures were designed (CAM-THY) (8, 14, 15).

## MS and Thyroid Diseases

Multiple sclerosis is a human chronic inflammatory disease of the central nervous system supposed to be a Th1/Th17 type cell-mediated autoimmune disorder (16, 17). Studies aimed at evaluating whether there is an increased prevalence of autoimmune thyroid diseases (AITDs) in patients with MS as compared with healthy controls reported conflicting results. While early studies found an increased prevalence of AITD in patients with MS, more recent surveys reported rates which are consistent with the AITD prevalence in the general population (18–21). According to more recent views, an increased prevalence of AITD would be observed in family members of patients with MS (22). This is a rather intriguing and yet to be a fully elucidated observation (23, 24).

In addition to studies aimed at evaluating the prevalence of AITD in naive patients with MS, the occurrence of AITD, as a side effect of immunomodulatory treatments for MS, was extensively reported (25–27). At present, we know that treatment with interferon-β (IFN-β) increases the risk for worsening and/or *de novo* appearance of both thyroid autoimmunity and dysfunction. On the other hand, thyroid side effects were not observed following glatiramer acetate (GA) therapy, even in large series of patients with MS who were longitudinally followed for more than 10 years (11, 28, 29).

Specifically designed head-to-head clinical studies demonstrated a similar efficacy of IFN-β and GA, as assessed by their ability to prevent clinical relapses and disease progression in patients with MS (12). However, more effective pharmacologic agents are now available. Among them, Alemtuzumab is currently regarded as an effective second-line treatment in patients with highly active RRMS (1, 2, 13).

## TSH-Receptor (TSH-R) as a Major Autoantigen in Graves’ Disease

Graves’ disease (GD), also referred to as toxic diffuse goiter, is commonly regarded as an autoimmune organ-specific disease (30–32). The presence of extra-thyroid manifestations, such as Graves’ orbitopathy and pretibial myxedema, apparently contradicts this classification, which is, however, justified by the TSH-R being a common antigen shared by the thyroid gland and by extra-thyroid tissues (31). The TSH-R, a G-protein coupled receptor with seven transmembrane-spanning domains and a large extracellular portion, is expressed primarily on the surface of thyroid follicular cells, but it is also present in adipocytes, fibroblast, bone cells, and other sites including the heart (33). TSH-R Antiboides (TRAb) encompass stimulating, blocking, and neutral antibodies. In patiens with GD, TRAb mainly have thyroid-stimulating activity (TSAb), which results in hyperthyroidism and goiter formation, TSAb bind only the naturally conformed TSH-R and induce cyclic AMP generation, thyroid cell proliferation, and thyroid hormone synthesis and secretion (34, 35). More rarely, and less functionally dominant, TRAb with thyroid blocking activity have been described in patients with GD (36).

Immune system abnormalities in GD are represented by TSH-R-reactive B cells, which escape deletion and eventually present this thyroid autoantigen to T cells. When activated, T cells secrete several pro-inflammatory cytokines and chemokines (37, 38). Hence both B cells and T cells play a central role in perpetuating the autoimmune cascade in GD (11, 37).

## AITDs and Alemtuzumab

Among several autoimmune conditions, which have been reported to occur following Alemtuzumab treatment, the present review will focus on GD, the most frequently observed one. The occurrence of GD in Alemtuzumab-treated patients with secondary progressive MS was first described in 1999 (39). In this early report, a third of MS patients (9/27) receiving the anti-CD52 monoclonal Ab developed GD, with circulating TRAb and hyperthyroidism (39). Besides being the first description, the study by Coles et al. is of great interest, as it clearly shows that GD (and its humoral marker, TRAb) is the most prevalent form of AITD occurring in MS patients treated with Alemtuzumab (39). By contrast, in MS patients, treated with other immunomodulatory therapies (i.e., IFN-β), the most prevalent side effect is represented by euthyroid or hypothyroid autoimmune thyroiditis (26, 27, 40). Thus, a first crucial difference between IFN-β and Alemtuzumab was already evident: they elicited different autoimmune reactions driving the onset of Hashimoto’s thyroiditis and GD, respectively.

Subsequent studies, investigating the efficacy and safety of Alemtuzumab therapy in patients with RRMS, confirmed GD as the main autoimmune sequela of this immunomodulatory treatment. In 2008, Coles et al. published a phase 2 clinical trial (CAMMS223) in which 334 patients with RRMS were randomized to receive either IFN-β-1a three times/week or annual cycles of Alemtuzumab (either 12 or 24 mg/day) for 3 years (41). Among other (rare) autoimmune side effects, such as trombocytopenic purpura and glomerulonephritis due to autoantibodies binding the glomerular basal membrane, they reported a significantly higher rate of thyroid autoimmunity in patients treated with Alemtuzumab as opposed to those receiving IFN-β-1a (22.7 versus 2.8%, respectively) (41). In 2012, two phase-3 trials also reported the occurrence of mild to moderate thyroid dysfunction in nearly 18% of RRMS patients treated with Alemtuzumab (1, 2).

The early series of patients included in the CAMMS223 study (2) was further investigated with the specific aim of evaluating thyroid side effects of Alemtuzumab therapy (42). Daniels et al. prolonged the surveillance period of these patients up to a median time of 57.3 months and a maximum of 80.6 months (42). They confirmed that thyroid dysfunctions more frequently occurred in patients treated with Alemtuzumab as compared to those receiving IFN-β-1a. In particular, 34% of patients treated with Alemtuzumab developed thyroid dysfunctions (39% receiving 12 mg and 29% receiving 24 mg) as compared with a 6.5% rate in those treated with IFN-β-1a. As shown in Table 1, in the Alemtuzumab treatment group, GD was the most prevalent condition, being experienced by nearly 23% of patients (42). Hypothyroidism was observed in 7.4% patients and destructive thyroiditis with thyrotoxicosis in 4.2% of patients (42).

**Table 1:** Thyroid dysfunction during Alemtuzumab treatment in patients with multiple sclerosis (MS).

Patients and dysfunctions	(%)
**Patients with thyroid dysfunction in the 216 patients cohort**	**34**
Graves’ hyperthyroidism Hypothyroidism Destructive thyroiditis	22.4 7.4 4.2
**Type of dysfunction in the 73 affected patients**	
Graves’ hyperthyroidism Hypothyroidism Destructive thryoiditis Unknown	65.8 20.5 10.3 1.4
**Number of events of thyroid dysfunction between the 102 recorded events**	
Single Multiple	70.0 30.0
**Type of event among the 102 recorded events**	
Graves’ hyperthyroidism ◦ Overt ◦ Subclinical Hypothyroidism Destructive thryoiditis Unknown Graves’ orbitophaty	58.8 81.7 18.3 29.4 9.8 2.0 6.0

*Results of the CAMMS223 study as categorized by Daniels et al. (42)*.

Some clinical peculiarities of these patients are worth noting. The first episode of thyroid dysfunction was observed starting from the first year after Alemtuzumab administration. Afterward, the episodes’ prevalence progressively increased each year for the first 3 years (from 4.6 to 16.1%) with a subsequent decrease in the following 4 years (from 11.3 to 5.9%). There was a higher than expected prevalence (52.7%) of patients in whom Graves’ hyperthyroidism (either overt or subclinical) was spontaneously reverted to hypothyroidism (either overt or subclinical) (Table 2). An unusual frequency of patients converting from hyperthyroidism to hypothyroidism and *vice versa* was also observed. Importantly, the conversion from hyperthyroidism to hypothyroidism was accompanied by the occurrence of TRAb in nearly 77% of patients (Table 3). This observation strongly suggests that the conversion from hyperthyroidism to hypothyroidism was likely due to a shift in the ratio between stimulating and blocking TRAbs. In the routine endocrine practice of sporadic GD, the transition from hyperthyroidism to hypothyroidism is a unusual event, which is mainly observed several years after a successful course of antithyroid drugs and is rarely accompanied by TRAb positivity (43). Taken together, these data indicate that the immune reconstitution occurring after Alemtuzumab treatment is mainly humoral, being directed to the TSH-R as a major autoantigen.

**Table 2:** Treatment and outcome of Graves’ disease developing after Alemtuzumab therapy.

	(%)
Therapy of overt hyperthyroidism in Graves’ disease	
Antithyroid drugs alone Antithyroid drugs + radiometabolic therapy (131-I) Radiometabolic therapy alone (131-I) Surgery (total thyroidectomy)	40.1 12.2 6.1 4.0
Outcome of *overt* hyperthyroidism in Graves’ disease	
Spontaneous resolution with: – Euthyroidism – Hypothyroidism	36.7 20.4 16.3
Therapy of *subclinical* hyperthyroidism	
Antithyroid drugs alone	36.4
Outcome of *subclinical* hyperthyroidism in Graves’ disease	63.6
Spontaneous resolution Subclinical hypothyroidism Overt hypothyroidism Unknown	18.2 18.2 18.2 9.1

*Data elaborated from Daniels et al. (42)*.

**Table 3:** TSH-receptor antibodies (TRAb) and thyroid dysfunction during Alemtuzumab treatment.

Patients and TRAb	(%)
Positive TRAb at baseline	0.0
*De novo* positive TRAb	38.0
Thyroid dysfunction and TRAb status	
Positive TRAb	70.0
Hyperthyroidism and TRAb status	
Positive TRAb	84.7
Graves’ disease and TRAb status	
Positive TRAb	100
Hypothyroidism and TRAb status	
Positive TRAb	76.7

*Data elaborated from Daniels et al. (42)*.

Risk factors for the development of Alemtuzumab-induced GD were a family history of thyroid diseases, female sex, younger age, smoking habit, lower administered dose of the monoclonal Ab, and pretretment postivity for thyroid peroxidase (TPO) antibody (Ab). However, TPOAb had a minor relevance as a risk factor, due to the low frequency of pre-teratment positive results for this autoimmune marker (Table 4). Daniels et al. found that only 16/206 (8%) patients were positive for TPOAb at baseline. Among them, the prevalence of thyroid dysfunction after Alentuzumab treatment was 69%, a much higher rate than the 31% one observed in patients who were TPOAb negative at baseline (44). However, the majority (85%) of patients developing a thyroid disorder were negative for TPOAb before Alentuzumab treatment. Therefore, regardless of the pretreatment TPOAb status, patients may develop a thyroid disfunction and should have thyroid function tests performed periodically (41).

**Table 4:** TPOAb and thyroid dysfunction during Alemtuzumab treatment.

Patients and TPOAb	(%)
**Patients with postive TPOAb at baseline**	**8.0**
**Patients with negative TPOAb at baseline who develop thyroid dysfunction**	**31.0**
**Patients with postive TPOAb at baseline who develop thyroid dysfunction**	**69.0**
Graves’ hyperthyroidism Hypothyroidism Destructive thyroiditis Euthyroidism	31.2 25.0 12.5 31.2
**Positive TPOAb at the time of thyroid dysfunction**	**69.8**
Positive TPOAb at baseline *De novo* positive TPOAb Persistently positive TPOAb	15.1 54.8 30.1
**De novo positive TPOAb without thyroid dysfunction**	**10.5**

*Data elaborated from Daniels et al. (42)*.

The remission rate of Alemtuzumab-induced Graves’ hyperthyroidism, either spontaneous or after antithyroid drug treatment (Table 2), was also higher (78%) than what commonly observed in the sporadic form of the disease (32).

In a futher observational cohort study, Tuohy et al. (45) re-evaluated 87 patients with RRMS who had been treated with Alemtuzumab in investigator-led studies in Cambridge from 1999 to 2012. This series included 67 patients of the CAMMS224 trial (18) and 20 of the SM3 trial (46). Among the 86 patients who completed the study, 35 (41%) developed a thyroid dysfunction, which was diagnosed as Graves’ hyperthyroidism in 22 (63%) and as hypothyroidism with positive tests for TPOAb in 12 (34%) of them. The main limitation of this study is that TRAb were not measured. At the present, large series studies aimed at evaluating the occurrence of thyroid dysfunctions in RRMS patients treated with Alemtuzumab reported a prevalence ranging from 16.7 to 41% (Table 5). The remaining published studies on the occurrence of GD in Alemtuzumab-treated patients mainly consist of single case or small series reports, which are summarized in Table 6. In the majority of these reports, patients developing AITD were taking Alemtuzumab for a RRMS (47–50). However, reconstitution GD was also described in patients receiving Alemtuzumab for other clinical conditions. The development of Graves’ hyperthyroidism was described by Walsh et al. in 11% of patients treated with Alemtuzumab for vasculitis (51). Other reports include (i) a young kidney transplant recipient who developed GD 4 years after Alemtuzumab treatment (52) and (ii) three pediatric cases of thyroid autoimmune diseases in patients receiving Alemtuzumab after hematopoietic cell transplantation (53).

**Table 5:** Studies reporting Alemtuzumab-related thyroid dysfunction.

Reference	No. of patients	No. of patients who developed thyroid dysfunctions	Thyroid function
Coles et al. (39)	27	9/27 (33%)	Hyperthyroidism: 9 (33%)^a^
Coles et al. (41)	216^a^	49/216 (22.7%)	Hyperthyroidism: 32 (14.8%)
			Hypothyroidism: 15 (6.9%)
Coles et al. (2)	596	100/596 (16.7%)	Hyperthyroidism: 28 (4.7%)
			Hypothyroidism: 31 (5.2%)
Cohen et al. (1)	376	68/376 (18%)	Hyperthyroidism: 28 (7%)
			Hypothyroidism: 18 (5%)
Tuohy et al. (45)	87	35/87 (41%)	Hyperthyroidism: 22 (25.3%)
			Hypothyroidism: 12 (13.8%)

*^a^One patient developed hypothyroidism before shifting to hyperthyroidism*.

**Table 6:** Case reports of patients developing autoimmune thyroid diseases following Alemtuzumab administration.

Reference	Case(s)	Disease	Thyroid function	Thyroid Ab	Treatment
Kirk et al. (52)	1 (F)	Kidney transplantation	Hyperthyroidism	TSH-RAb+	Carbimazole
				TgAb n/a	
				TPOAb n/a	

Aranha et al. (47)	1 (F)	Multiple sclerosis (MS)	Hyperthyroidism	TSH-RAb+ TgAb+ TPOAb+	Carbimazole, thyroidectomy

	2 (F)	MS	Hyperthyroidism	TSH-RAb+ TgAb− TPOAb−	Carbimazole, thyroidectomy

	3 (F)	MS	Hyperthyroidism, then hypothyroidism	TSH-RAb+ TgAb n/a TPOAb n/a	Carbimazole, then levothyroxine

	4 (F)	MS	Hyperthyroidism	TSH-RAb+ TgAb+ TPOAb+	Carbimazole

Tsourdi et al. (48)	1 (M)	MS	Hyperthyroidism	TSH-RAb+ TgAb+ TPOAb+	Thiamazole

	2 (F)	MS	Hyperthyroidism	TSH-RAb+ TgAb+ TPOAb+	Thiamazole, thyroidectomy

	3 (F)	MS	Hyperthyroidism	TSH-RAb+ TgAb+ TPOAb–	Thiamazole, thyroidectomy

	4 (M)	MS	Hyperthyroidism	TSH-RAb+ TgAb− TPOAb+	Thiamazole, thyroidectomy

	5 (F)	MS	Mild hyperthyroidism	TSH-RAb+ TgAb+ TPOAb+	No therapy

Williams et al. (53)	1 (M)	Hematopoietic cell transplantation	Hypothyroidism	TSH-RAb n/a TgAb+ TPOAb+	Levothyroxine

	2 (M)	Hematopoietic cell transplantation	Hyperthyroidism	TSH-RAb− TgAb+ TPOAb+	Methimazole

	3 (M)	Hematopoietic cell transplantation	Hyperthyroidism	TSH-RAb− TgAb+ TPOAb+	Methimazole

Mahzari et al. (49)	1 (M)	MS	Hyperthyroidism, then hypothyroidism	TSH-RAb n/a TgAb n/a TPOAb+	Propylthiouracil, then levothyroxine

	2 (F)	MS	Hyperthyroidism, then hypothyroidism	TSH-RAb n/a TgAb n/a TPOAb+	No therapy, then levothyroxine

	3 (F)	MS	Hyperthyroidism	TSH-RAb n/a TgAb n/a TPOAb+	Methimazole

	4 (F)	MS	Mild hyperthyroidism, then hypothyroidism	TSH-RAb n/a TgAb n/a TPOAb+	No therapy, then levothyroxine

Obermann et al. (50)	1 (M)	MS	Hyperthyroidism	TSH-RAb+ TgAb+ TPOAb+	Carbimazole

	2 (M)	MS	Subclinical hypothyroidism	TSH-RAb− TgAb+ TPOAb+	No therapy

At variance with all the above described studies, the development of immune reconstition GD was not observed in a large series of patients with rheumatoid arthritis treated with Alemtuzumab (54). These negative findings support the view that patients with MS bear a higher intrinsic risk for the development of Alemtuzumab-related thyroid dysfunctions. Further support to the above statement stems from the notion that while Alemtuzumab is increasingly prescribed in chronic lymphocytic leukemia, no case of Graves’ hyperthyroidism has been reported in these patients.

## Final Remarks

Reconstitution GD may occur during the recovery phase of Alemtuzumab-induced CD52 cells depletion. Because reconstitution autoimmunity is more frequently related to autoantibody-mediated diseases rather than to destructive, Th1-mediated disorders (i.e., Hashimoto’s thyroiditis), it is not surprising that GD is the most commonly reported side effects of Alemtuzumab treatment.

The reason why, as compared with patients bearing other clinical conditions, those with MS carry a higher risk for the development of GD after Alemtuzumab treatment remains unknown. Genetic factors and/or specific clinical aspects of MS, such as the cytokine/chemokine milieu and/or the RR clinical course, might play a role, but there is still no definite proof at this regard.

From a clinical point of view, there are peculiar aspects of Alemtuzumab-induced GD. First, hyperthyroid patients have an unusualy high rate of spontaneous shift to hypothyroidism. This shift is supposed to result from a change from stimulating to blocking TRAb. Second, the remission rate of Graves’ hyperthyroidism, both spontaneous and after antithyroid drugs, is unexpectedly high, suggesting a less aggressive disease (14). This observation implies that antithyroid drugs should be the first-line treatment in patients with Alemtuzumab-induced Graves’ hyperthyroidism.

## Author Contributions

LC, MR, MaM, PL, VC, and MiM designed the study and reviewed the literature on thyroid side effects of Alemtuzumab. LC and MR wrote the manuscript. All the authors revised the paper and approved the final edition.

## Conflict of Interest Statement

The authors declare that the research was conducted in the absence of any commercial or financial relationships that could be construed as a potential conflict of interest.
